# Supplementary material for: Preparation and Properties of Salecan–Soy Protein Isolate Composite Hydrogel Induced by Thermal Treatment and Transglutaminase
Source: Int J Mol Sci. 2022 Aug 20;23(16):9383. doi: 10.3390/ijms23169383 (PMC9409434; doi:10.3390/ijms23169383)
Supplement: Supplementary file 1 [file ijms-23-09383-s001.zip › ijms-1839509-supplementary.pdf]

Supporting Information to the manuscript

Preparation and Properties of Salecan-Soy Protein Isolate Composite Hydrogel Induced by Thermal Treatment and Transglutaminase

Jing Gan [1,†], Lirong Sun [2,†], Chenxia Guan [2], Teng Ren [2], Qinling Zhang [2], Shihui Pan [2], Qian Zhang[2] and Hao Chen [2,*]

[1] College of Life Science, Yantai University, NO.30 Qingquan Road, Laishan Strict, Yantai, 264000, China

[2] Marine college, Shandong University, NO.180 Wenhua West Road, Gao Strict, Weihai 264209,China

* Correspondence: chenhao_2016@sdu.edu.cn; Tel.:+86-13366061833

† These authors contributed equally to this work.

Table S1. Summary of the raw materials and characteristics of Protein- Polysaccharide composite hydrogel.

Materials	Gelling methods	Properties and Functions	Reference
Gelatin/Carrageenan	Glutaraldehyde crosslinking	Good gel strength, potential barrier for microbes	[1]
Chitosan/Gelatin	EDC crosslinking	Biodegradability, fast gelation, reducing cecum-abdomen adhesion formation	[2]
Silk fibroin/ Tyramine-modified hyaluronic acid	Laccase-catalyzed	High structural stability, improved mechanical properties	[3]
Diosgenin-nanocellulose/ Gelatin	Genipin crosslinking	good swelling capacity, high gel yield, excellent antibacterial effect	[4]
Flammulina velutipes polysaccharide/ soy protein	Thermal treatment	uniform and dense microstructure, low viscosity, higher water-binding ability, good 3D printing quality	[5]

2. Results and Discussions

2.9. Thermal stability of Sal-SPI composite hydrogel

The relationship curves of the weight loss of Sal, SPI, and Sal-SPI composite hydrogels as a function of temperature are shown in Figure S1(a) and Figure S1(b). All samples show a slight weight loss near 100°C, which may be due to water evaporating from the surface of the samples. Sal loses roughly 60.4% of its weight in the range of 288~600 °C, which corresponds to the break of the depolymerized side chain of the C=O and C=C bonds of the sugar ring and the dehydration degradation of the main chain[6]. For SPI, there are two stages of decomposition: at 204~383 °C, the weight loss is 50.1%, which can be attributed to the breakage of the side chain groups including NH_2 and COOH, as well as the destruction of the hydrogel network; at 384~583 °C, the weight loss is 12.4% which

could be due to the breakage of the protein backbone. The thermogravimetric analysis curve of Sal-SPI composite hydrogel showed a similar tendency to that of a single SPI hydrogel. As shown in Figure S1(b), the maximum mass loss rate temperature (Tmax) of Sal-SPI composite hydrogel is 312 °C greater than that of a single SPI hydrogel (302 °C). This result demonstrates that the thermal stability of Sal-SPI composite hydrogel improved after the introduction of Sal. With the help of hydrogen bonds, electrostatic interactions, and other intermolecular interaction forces, SPI and Sal chains could form a denser network structure, increasing the thermal stability of the composite hydrogel.

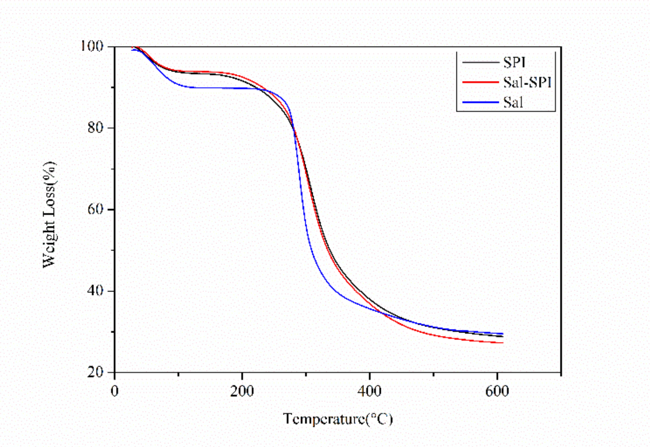

(a)

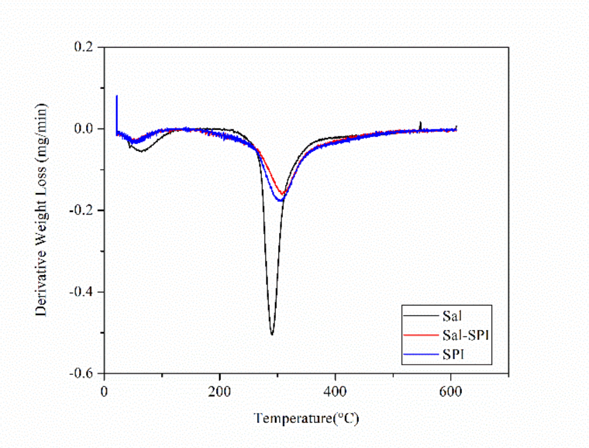

(b)

Figure S1. (a) Thermogravimetric Analysis (TGA)of Sal, SPI, and Sal-SPI composite

hydrogel; (b) DTGA of Sal, SPI, and Sal-SPI composite hydrogel.

3. Materials and Methods

3.7. Thermogravimetric analysis (TGA)

The lyophilized Sal single hydrogel, SPI single hydrogel, and Sal-SPI composite samples (about 3 mg) were weighed and placed in a thermogravimetric analyzer (METTLER, Switzerland) for detection. The testing temperature were ranging from 25 to 600°C, the nitrogen purge speed was 100 mL/min, and the heating rate was 10°C/min [7].

References

1. Haima, J. S.; Nair, S. N.; Juliet, S.; Nisha, A. R.; Dhanushkrishna, B. N., Synthesis and characterisation of glutaraldehyde cross-linked κ-carrageenan-gelatin hydrogel. *J. pharmacogn. phytochem* **2021,** 10, (1), 459-463.
2. Lin, L. X.; Luo, J. W.; Yuan, F.; Zhang, H. H.; Ye, C. Q.; Zhang, P.; Sun, Y. L., In situ cross-linking carbodiimide-modified chitosan hydrogel for postoperative adhesion prevention in a rat model. *Mater Sci Eng C Mater Biol Appl* **2017,** 81, 380-385.
3. Wang, L.; Xu, B.; Nong, Y.; Wang, P.; Yu, Y.; Deng, C.; Yuan, J.; Wang, Q., Laccase-mediated construction of flexible double-network hydrogels based on silk fibroin and tyramine-modified hyaluronic acid. *Int J Biol Macromol* **2020,** 160, 795-805.
4. Ilkar Erdagi, S.; Asabuwa Ngwabebhoh, F.; Yildiz, U., Genipin crosslinked gelatin-diosgenin-nanocellulose hydrogels for potential wound dressing and healing applications. *Int J Biol Macromol* **2020,** 149, 651-663.
5. Pan, H.; Pei, F.; Ma, G.; Ma, N.; Zhong, L.; Zhao, L.; Hu, Q., 3D printing properties of Flammulina velutipes polysaccharide-soy protein complex hydrogels. *J Food Eng* **2022,** 334.
6. Hu, X.; Wang, Y.; Zhang, L.; Xu, M., Construction of self-assembled polyelectrolyte complex hydrogel based on oppositely charged polysaccharides for sustained delivery of green tea polyphenols. *Food Chem.* **2020,** 306, 125632.
7. Qi, X.; Su, T.; Zhang, M.; Tong, X.; Pan, W.; Zeng, Q.; Shen, J., Sustainable, flexible and biocompatible hydrogels derived from microbial polysaccharides with tailorable structures for tissue engineering. *Carbohydr. Polym.* **2020,** 237, 116160.
